# Prediction of biological age by morphological staging of sarcopenia in *Caenorhabditis elegans*

**DOI:** 10.1242/dmm.049169

**Published:** 2021-11-30

**Authors:** Ineke Dhondt, Clara Verschuuren, Aleksandra Zečić, Tim Loier, Bart P. Braeckman, Winnok H. De Vos

**Affiliations:** 1 Biology Department, Ghent University, K.L. Ledeganckstraat 35, B-9000 Ghent, Belgium; 2 Laboratory of Cell Biology and Histology, Department of Veterinary Sciences, University of Antwerp, 2610 Antwerp, Belgium

**Keywords:** Sarcopenia, *C. elegans*, Ageing, Healthspan, Image analysis, Phenotype prediction

## Abstract

Sarcopenia encompasses a progressive decline in muscle quantity and quality. Given its close association with ageing, it may represent a valuable healthspan marker. The commonalities with human muscle structure and facile visualization possibilities make *Caenorhabditis elegans* an attractive model for studying the relationship between sarcopenia and healthspan. However, classical visual assessment of muscle architecture is subjective and has low throughput. To resolve this, we have developed an image analysis pipeline for the quantification of muscle integrity in confocal microscopy images from a cohort of ageing myosin::GFP reporter worms. We extracted a variety of morphological descriptors and found a subset to scale linearly with age. This allowed establishing a linear model that predicts biological age from a morphological muscle signature. To validate the model, we evaluated muscle architecture in long-lived worms that are known to experience delayed sarcopenia by targeted knockdown of the *daf-2* gene. We conclude that quantitative microscopy allows for staging sarcopenia in *C. elegans* and may foster the development of image-based screens in this model organism to identify modulators that mitigate age-related muscle frailty and thus improve healthspan.

## Abstract (precis)

**Summary:** A tool for quantitative image analysis of muscle deterioration that allows predicting healthspan in the nematode model *Caenorhabditis elegans* and may lead to the first *C. elegans*-based high-throughput sarcopenia screening platform.

## INTRODUCTION

Ageing is a complex phenomenon, which can be defined as a progressive deterioration of cell and tissue functions in living organisms with age (Gems, 2014). For decades, biogerontologists exclusively focused on the determinants of lifespan with an eye on its extension in diverse organisms. However, rather than just extending lifespan per se, it is becoming increasingly clear that it is more desirable to improve the quality of life by limiting the risk for frailty and disease at advanced age (Olshansky, 2018). Hence, extending healthspan, the life period in which one is functionally independent and free from serious disease, has now become a central theme of modern biogerontology (Luyten et al., 2016; Rattan, 2018). A key component of human late-life frailty is sarcopenia, a progressive decline in muscle quantity and quality (Evans et al., 2010; Fulop et al., 2010). A delayed onset of sarcopenia may thus represent a valuable biomarker of extended healthspan. Owing to its genetic amenability and short lifespan, *Caenorhabditis elegans* has become an invaluable model organism for the study of ageing. As its soma is post-mitotic and transparent, the progression of several age-related pathologies can easily be followed *in vivo*.

The musculature of *C. elegans* includes both striated and non-striated muscle cells. Striated muscle cells are limited to the body wall, whereas non-striated cells are more widely distributed in the body and can be found in structures such as the pharynx and intestine-associated and gonad-associated muscles. The 95 striated body wall muscle cells are organized into two ventral and two dorsal muscle quadrants that stretch longitudinally from head to tail. Each body wall muscle cell is attached to the underlying hypodermis and neighbouring muscle cells. Rather than being innervated by motoneuron axons, *C. elegans* body wall muscle cells contain muscle arms that reach out to the nervous system for innervation (Altun and Hall, 2009).

Ageing *C. elegans* experience gradual, progressive muscle deterioration, resembling human sarcopenia. In young nematodes, body wall muscle myofilaments are well organized in a tight, parallel manner, whereas in older animals they show progressive disorganization, irregular orientation, breaks and abnormal bends (Herndon et al., 2002). The ease with which muscle structure can be visualized *in vivo* and the similarity of its muscle microanatomy to that of man make *C. elegans* a strong model for studying sarcopenia (Christian and Benian, 2020; Herndon et al., 2002; Meissner et al., 2009). Additionally, the lack of muscle stem cells in this nematode model allows focusing on muscle deterioration during ageing without the confounding influence of muscle regeneration (Christian and Benian, 2020). Worm myofilament organization is often studied by visual assessment of a reporter strain expressing fluorescent markers such as GFP-tagged myosin heavy chain (MHC) A (MYO-3) in the body wall muscle cells (Campagnola et al., 2002; Glenn et al., 2004). Former studies relied almost exclusively on manual scoring of myofilament organization and the appearance of muscle defects (Ben-Zvi et al., 2009; Brehme et al., 2014; Glenn et al., 2004; Herndon et al., 2002; Karady et al., 2013; Lamarche et al., 2018; Meissner et al., 2009, 2011). Such quantifications are prone to observer bias and are not very sensitive. Hence, we developed an objective analysis tool for the detailed quantification of muscle integrity in myosin::GFP reporter strains. In order to stage the severity of sarcopenia in ageing muscle cells, we extracted a variety of morphological descriptors (features) of individual myofilaments within the muscle cell using an automated image-processing pipeline. We found that a subset of features scaled linearly with age, allowing us to establish a linear model to predict biological age from a morphological muscle signature. To prove its potential for screening, we validated our model on a biologically relevant mutant displaying delayed sarcopenia (Depuydt et al., 2013; Herndon et al., 2002; Wang et al., 2019).

## RESULTS

### Sarcopenia occurs progressively with age in *C. elegans*

**Fig. 1.**
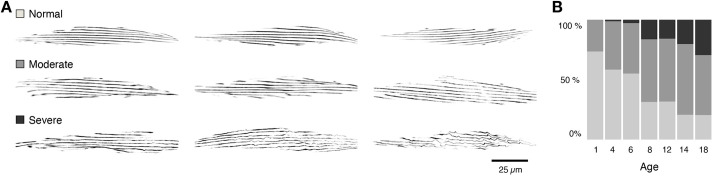
**Age-dependent defects in structural organization of the *C. elegans* body wall muscle.** Representative cropped confocal images of individual *C. elegans* muscle cells according to three qualitative classes of muscle deterioration. (B) Blinded, manual scoring of randomized images shows the progressive loss of myofilament organization with age, as evidenced by the decrease in normal and increase in moderate to severe phenotypes.

Muscle deterioration is a common pathology observed in old animals (Christian and Benian, 2020). To document its penetrance and evolution in *C. elegans*, we set out to score the muscle phenotype with increasing age. To evaluate muscle architecture *in vivo*, we used a transgenic *myo-3*::GFP reporter strain, which expresses GFP-tagged MHCA in the body wall muscle cells. Confocal images were collected throughout the adult lifespan (day 1 to day 18) of four independent worm populations. At set time points, several muscle cells (*n*_muscle_=2-4) of multiple worms (*n*_worm_=5-12) were visualized using consistent acquisition settings (Table 1). As expected, and in line with earlier findings (Herndon et al., 2002), we observed a marked change in the organization of muscle cells with advancing age, indicative of ensuing sarcopenia. Young muscle cells typically displayed a tightly organized architecture of parallel oriented, thick myofilaments, whereas muscle cells of later life stages (day 8 to day 18), more frequently displayed bends or breaks in individual myofibres, leading to a less aligned pattern (Fig. 1A). To gain a first qualitative impression of the age dependence, we manually attributed a score to the muscle phenotype based on a visual assessment. We thereby discriminated three phenotypes, representing either a healthy well-organized cell, a moderately disorganized cell or a severely distorted muscle cell. To avoid bias, scores were assigned in a blinded manner. Although most (75%) muscle cells indeed showed a normal, healthy phenotype at day 1, the fraction of moderately or severely disordered cells progressively increased with age (Fig. 1B). At day 14 and 18, ∼75% of the muscle cells were found to be at least moderately distorted. This shows that the degree of sarcopenia increases gradually with age and suggests that its severity may serve as a biomarker for biological age.

**Table 1.**
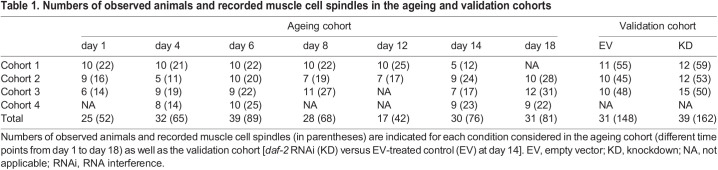
Numbers of observed animals and recorded muscle cell spindles in the ageing and validation cohorts

### Automated analysis reveals changing myofilament structure and heterogeneity with age

**Fig. 2.**
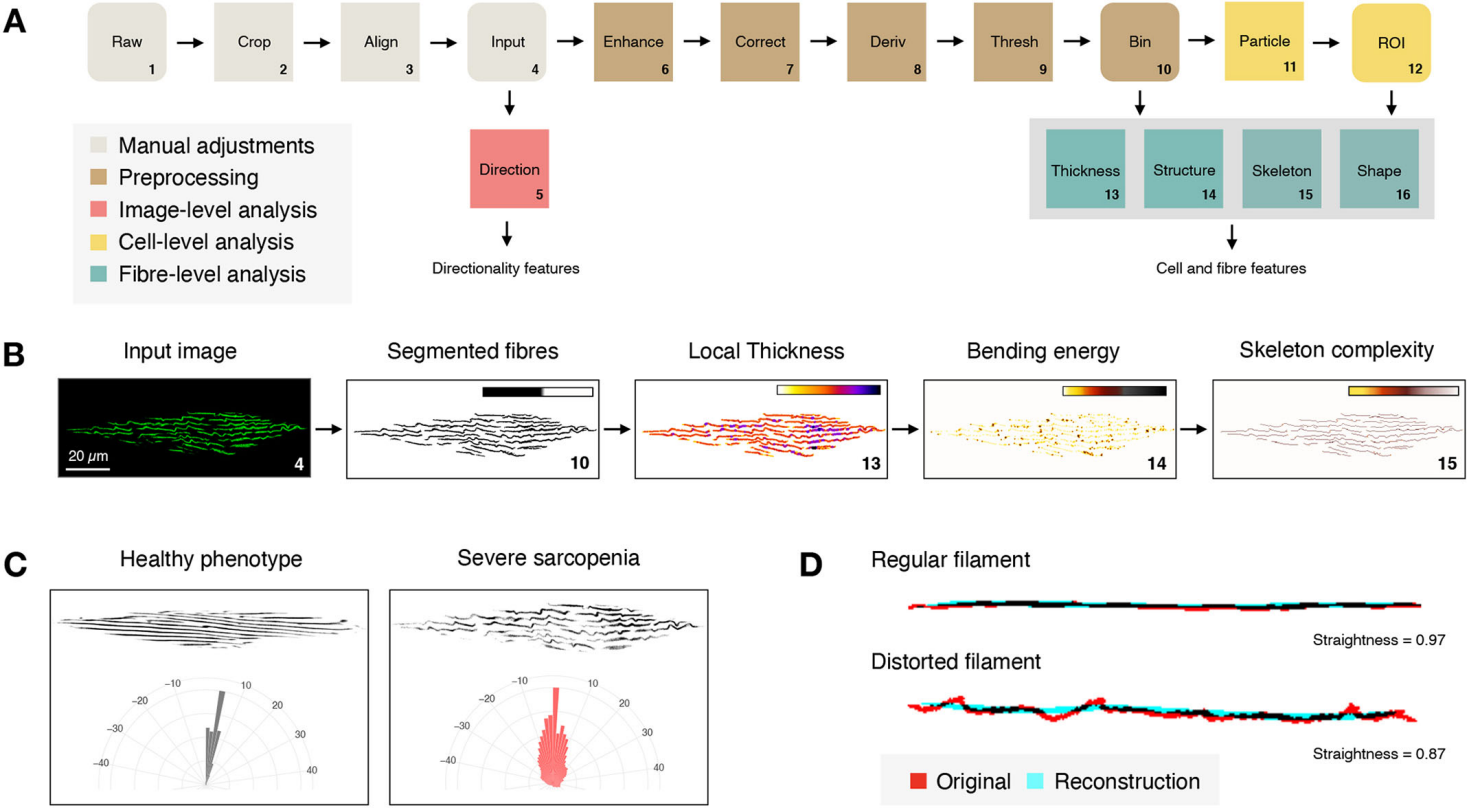
**Body wall muscle image analysis in *C. elegans*.** (A) Automated extraction of myofilament (organization) features using a custom-designed image-processing pipeline, consisting of manual pre-adjustment of the image (cropping and aligning individual cells from raw images resulting in the input image), pre-processing (local contrast enhancement, background correction, directional derivative and thresholding resulting in a binary image) and subsequent image analysis at different levels. The unprocessed input image is used for directionality analysis at the image level; the binary (segmented) image is used for detection of local thickness and structural variations as well as for skeletonization (complexity); and, after individual particle analysis, resulting regions of interest (ROI) are used for individual fibre measurements. (B) Illustration of interim results for an image of a severe sarcopenia phenotype (numbers correspond with steps in A). (C) Directional analysis in a healthy versus severely affected muscle cell. The orientation of myofilaments in the latter deviates significantly from the expected tight parallel myofilament organization observed in young animals, which is reflected in a broader distribution of orientation angles on a radar plot. (D) Fibre straightness is measured as the ratio of the perimeter of the original object (fibre) versus a reconstruction from a limited number of Fourier shape descriptors.

**Fig. 3.**
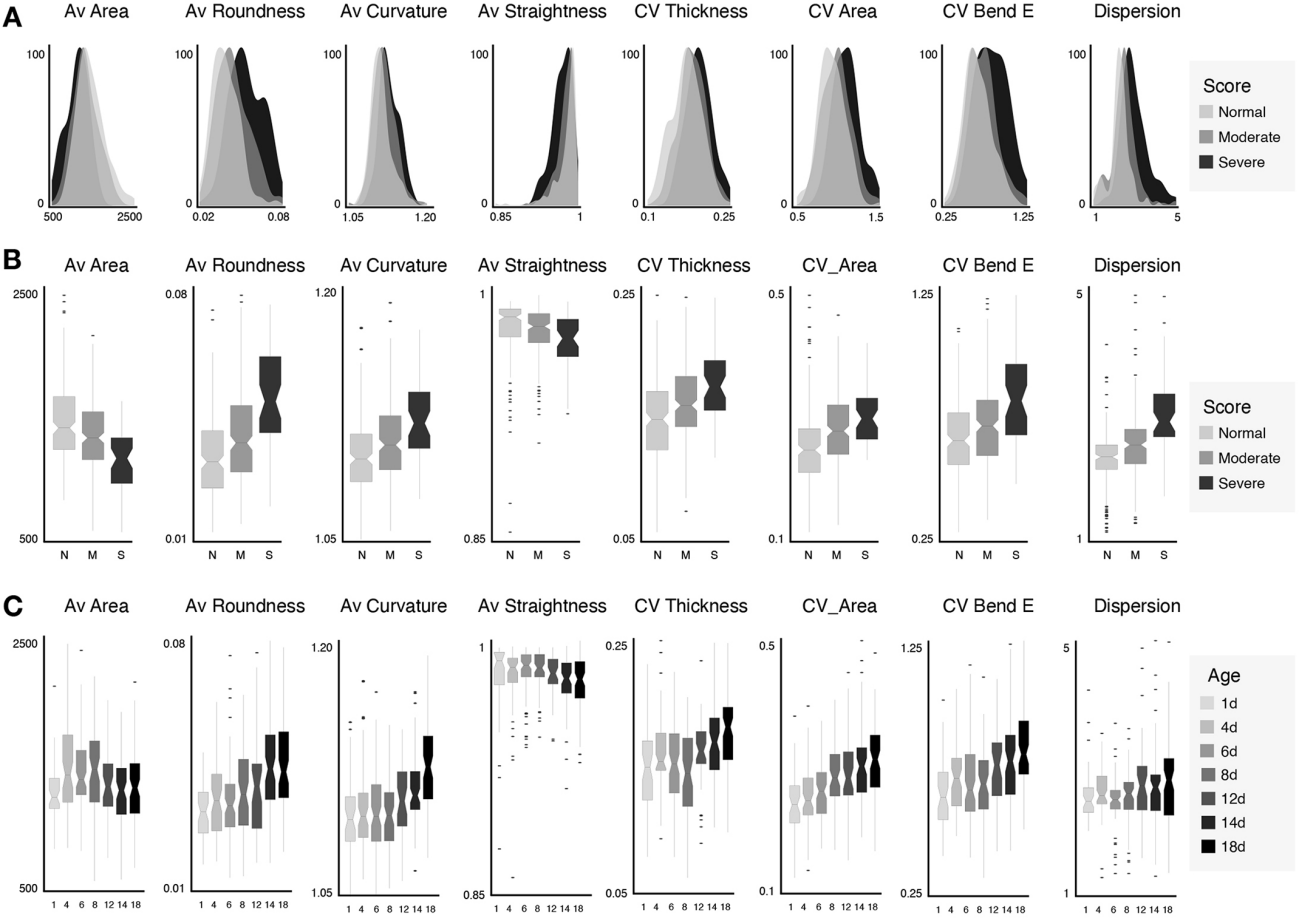
**Selected features describing morphological changes as a function of score and age.** (A) Histograms illustrating feature variability (per muscle cell) as a function of score. (B) Boxplots of the same features as in A per score. (C) Boxplots per age show similar trends. Av, average; CV, coefficient of variation.

Having established a correlation between age and the number of cells with distorted filaments, we next asked whether we could describe muscle organization more quantitatively so as to enable an objective, automated assessment. To this end, we developed an image analysis routine that extracts a variety of morphological descriptors (features) of individual muscle cells and their constituent myofilaments (Fig. 2, see Materials and Methods section). When inspecting the extracted feature space as a function of the manually attributed score, several clear trends could be observed (Fig. 3A,B; Fig. S1). Specifically, the fraction of smaller, more compact myofibres increased with severity score, as indicated by the lower values for average area and perimeter and larger values for average circularity and roundness. This may reflect the progressive myofibre fragmentation that accompanies sarcopenia. At the same time, the number of irregularly shaped, non-linear filaments increased with severity score, as indicated by the change in average curvature, straightness and bending energy. Not only the average value but also the variability of certain features varied with the attributed score. For example, the covariance of the filament thickness, area, bending energy and solidity increased with severity score, suggesting a larger fibre heterogeneity within more distorted cells. Finally, the orientation of individual fibres became more heterogeneous with sarcopenia score, as evidenced by the larger dispersion and lower amount of directionality information that could be explained by Gaussian fit to the dominant orientation. When plotting the exact same features as a function of age instead of score (Fig. 3C; Fig. S2A), very similar trends were observed, suggesting that these metrics also portray the penetrance of sarcopenia in a population of ageing worms.

### A morphological muscle signature can be used to predict biological age

**Fig. 4.**
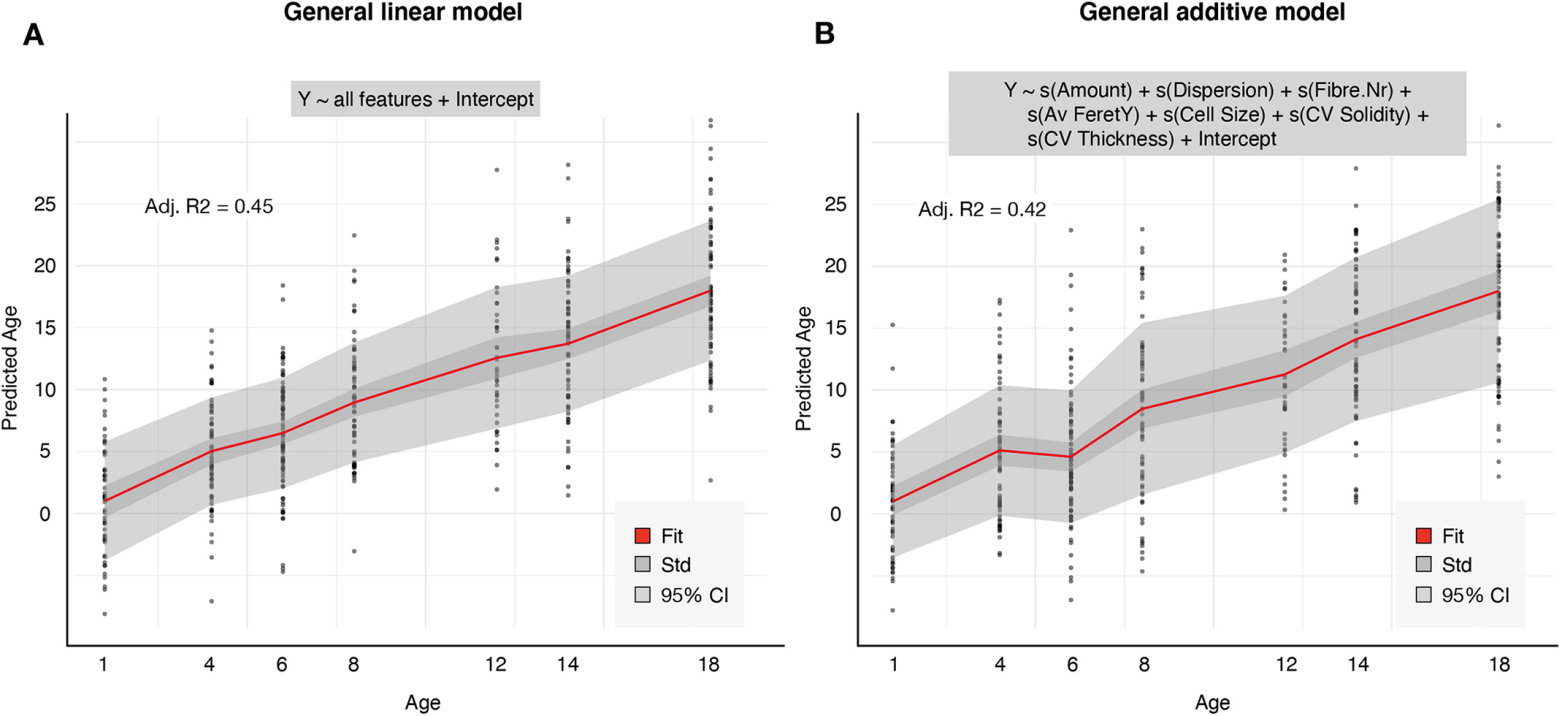
**Linear models predict biological age from muscle morphology in *C. elegans*.** (A) Linear regression on the complete dataset using all features. (B) General additive model with smoothing terms of seven selected features shows comparable performance. The average regression line is shown in red, the standard deviation ribbon in dark grey and 95% confidence interval in light grey.

Given the close resemblance between the feature distributions of severity score and organismal age, we hypothesized that the morphological features could inform on the biological age by providing an estimate of the degree of sarcopenia. In order to confirm this hypothesis, we first statistically queried the age dependence of the individual metrics. Univariate linear regression revealed that the magnitude of many features significantly scaled with age (46/60 features having a *P*-value <0.05, Fig. S2B), thus confirming the observed trends. However, the Pearson correlation coefficient (*R*) of individual comparisons was rather low, with a maximum value of *R*=0.41 for the average fibre number. We reasoned that a more inclusive model would perform better; therefore, we performed linear regression using all standardized features. The resulting linear model was able to account for a larger proportion of the variability as evidenced by an *R* of 0.72 (adjusted *R*^2^=0.45), despite the persistence of normal muscle fibres in older organisms (Fig. 4A).

Supported by these results, we wondered whether such a model could be used to predict age. To this end, we split our dataset into a training (2/3) and validation (1/3) set and established a complete linear model on the first to evaluate its performance on the latter. This revealed a comparable performance as for the full dataset [adjusted *R*^2^=0.43; root mean squared error (RMSE)=4.41]. Although the most complete model (using all features) will explain the largest proportion of the variability in the training set, it might not necessarily perform best on a separate validation set as it might be biased (overfitted) towards the former. To establish the most parsimonious model that performs well on both datasets, we therefore tried narrowing its feature space by setting cut-offs on multi-collinearity, either by removing variables with more than 60% correlation or variance inflation factors above 5 (Fig. S3). Doing so, we found that a model including only seven parameters (Average Amount, Average Dispersion, Average Fibre Number, Average Feret Y, CoV Solidity, Average Cell.Size, CoV_Thickness) performed equally well on the validation set as the full model (adjusted *R*^2^=0.45; RMSE=4.25). Introduction of smoothing terms (using a general additive model) slightly improved this further (adjusted *R*^2^=0.46; RMSE=4.26). For comparison, we calculated the same model for the full dataset (Fig. 4B). Thus, we conclude that a minimal signature of seven morphological descriptors, covering cell size, fibre directionality and fibre shape heterogeneity, can be used for estimating biological age in *C. elegans*. Yet, although it increased the interpretability, reducing the feature space did not significantly improve the prediction accuracy of the model.

### Morphological staging confirms sarcopenia delay in worms with reduced insulin/IGF1-like signalling

**Fig. 5.**
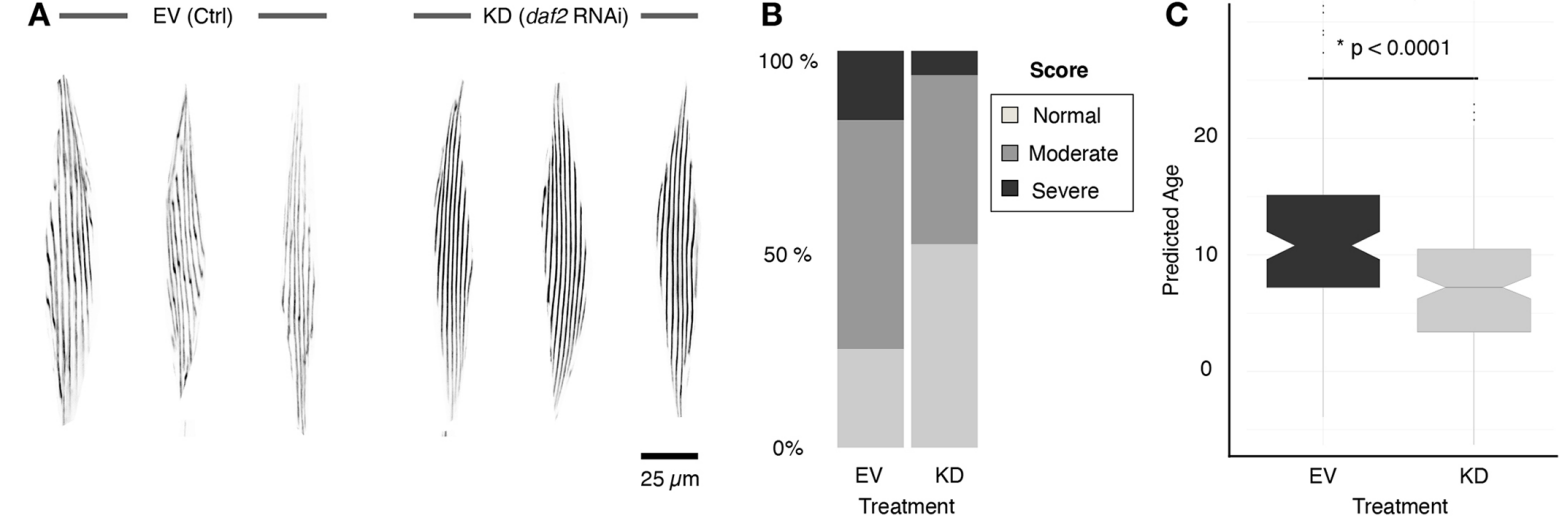
**A linear model predicts a younger biological age for worms with downregulation of *daf-2*.** (A) Representative cropped confocal images of body wall muscles from *daf-2* RNAi (KD) and EV-treated control (EV) worms at a chronological age of day 14. (B) Blinded, manual scoring of randomized images shows a higher number of normal and lower number of severe muscle phenotypes in KD worms. (C) Boxplot of the predicted age by the full linear model reveals a significant difference between KD and EV worms (Welch two-sample *t*-test). EV, empty vector, KD, knockdown; RNAi, RNA interference.

In a final step, we sought to benchmark the approach for predicting biological age in a physiologically validated model. Because reduced insulin/IGF1 signalling leads to lifespan extension in worms (Kenyon et al., 1993) and hypomorphic mutants of the insulin/IGF1-like receptor *daf-2* show increased healthspan (Podshivalova et al., 2017) including a delay in the onset of sarcopenia (Kashyap et al., 2012), we decided to use this model. We compared *daf-2* RNA interference (RNAi)-treated knockdown (KD) *myo-3*::GFP worms with empty vector (EV)-treated controls at advanced age (14 day) in three independent biological replicates. Visual scoring confirmed that the musculature was in a better condition in KD worms than in EV-treated controls (Fig. 5A), as evidenced by the reduction in severely affected muscle cells and concomitant increase in healthy-appearing (‘normal’) muscle cells (Fig. 5B). The animals were subjected to an identical image acquisition and analysis procedure as before, and the resulting standardized feature set was used as input for the established general linear model. Using the complete linear model, we observed a significant difference in the predicted biological age of KD worms compared to their EV counterparts (7 days versus 12 days, respectively; *P*=5.472×10^−8^, Welch two-sample *t*-test) (Fig. 5C). Hence, using a biological control, we provided an independent validation of the predictive value of the linear model for sarcopenia-based staging.

## DISCUSSION

In this work, we focused on the gradual microfilament deterioration in the *C. elegans* body wall muscle, an age-associated pathology that resembles human sarcopenia (Ben-Zvi et al., 2009; Christian and Benian, 2020; Herndon et al., 2002). Although worms only live 2-3 weeks, their body wall muscles showed clear structural disorder with increasing age. This vulnerability likely originates from the ‘built-for-life’ property of *C. elegans* muscle cells: they are non-replaceable and post-mitotic, and their myofilament proteins turn over extremely slowly (Dhondt et al., 2016, 2017). Although several studies have scored muscle defects in *C. elegans*, the large majority relied on manual scoring (Ben-Zvi et al., 2009; Brehme et al., 2014; Glenn et al., 2004; Herndon et al., 2002; Karady et al., 2013; Lamarche et al., 2018; Meissner et al., 2009, 2011). Only in one recent study were muscle cell area and myofilament length measured by image analysis to more objectively evaluate *C. elegans* body wall muscle integrity (Soh et al., 2020). However, this study focused on mutants with severe muscle defects, and although the two parameters may have had sufficient power to discriminate overt phenotypes, they do not have the resolution or sensitivity to capture the full extent of gradual morphological changes that take place during sarcopenia development. Hence, we introduced an unbiased approach to stage sarcopenia.

Having confirmed that the phenomenon is highly age dependent, we reasoned that its quantitative description could serve as a predictor of biological age and *in extenso* of healthspan, comparable to other *C. elegans* healthspan biomarkers, such as those related to muscle function and stress resistance (Hahm et al., 2015; Keith et al., 2014). Starting from an exhaustive extraction of morphological features from segmented muscle cells, a non-redundant set was identified that, together, describes the changes that are observed in muscle cells with biological age. The biological relevance of these changes can be explained and, in most cases, confirms earlier microscopic observations. Indeed, Herndon et al. (2002) found that aged worms have a loss of direction of individual sarcomeres, and the stochastic nature of worm sarcopenia described in that same paper is in accordance with the age-related increase in covariance we observed in several features. Of note, increased cellular heterogeneity appears to be a common feature in ageing, as was also established in a study of biophysical and molecular properties of human fibroblasts from an ageing cohort (Phillip et al., 2017). Myofilament breaks and bends are also often visible in old muscle cells and explain the smaller and more detected fibre entities. The increase in allover cell size is at odds with the notion that sarcopenia is characterized by reduction in muscle mass and concomitant flattening of the body wall muscle (Herndon et al., 2002).

Of note, a significant proportion of the *C. elegans* muscle cells retain a healthy phenotype at very advanced age. Although genotype and environment were held constant (within and between ageing cohorts), we observed large cell-to-cell variation in the degree of muscle deterioration within individuals, but also considerable inter-individual variability. This inherent variability was noted earlier as well (Herndon et al., 2002) and contributed to the uncertainty of our model. Notwithstanding the high number of ostensibly normal cells, the model was able to classify and predict biological age. Although increasing the number of sampled animals would most likely improve its accuracy to a certain extent, this is not easily manageable with a standard setting using individual microscopy slides. Hence, leveraging the approach to a high-throughput setting would demand a more controlled sampling, e.g. by organism-on-chip microscopy (de Cáceres et al., 2018; Kim et al., 2020; San-Miguel and Lu, 2013). Ideally, this could be combined with automated detection and delineation of muscle cells, which would make time-consuming manual segmentation steps unnecessary. However, owing to unclear outlines of body wall muscle cells, this would require more advanced detection algorithms, e.g. based on deep learning, than the currently available ones. Next to increasing the number of sampled worms to increase prediction accuracy, it may be worth investigating whether the variability in muscle phenotype can be reduced. In old worms, ∼25% of the myofilaments did not show obvious deterioration at the light-microscopic level, but it is conceivable that subthreshold, molecular damage may be present that is not readily visible under standard settings. A controlled paradigm to evoke mechanical stress, such as stimulated swimming in a shaking liquid culture over a fixed time period, may elicit the underlying molecular damage and result in a more uniform and outspoken myofilament phenotype in old worms.

In conclusion, we here present a tool that is capable of predicting *C. elegans* healthspan, based on myofilament features, as exemplified by our analysis of an insulin/IGF1-like signalling mutant. In its current form, this tool can be used downstream of the many high-throughput screens for genes, treatments and compounds that modulate healthspan in *C. elegans* (Bulterijs and Braeckman, 2020; Le et al., 2020; Luyten et al., 2016; Sayed et al., 2021). It objectively probes for the effect of such treatments on sarcopenia, an age-related pathology conserved from worms to humans. Further automation and upscaling of the protocol may lead to the first *C. elegans*-based high-throughput sarcopenia screening platform.

## MATERIALS AND METHODS

### *C. elegans* maintenance

In this study, we used the transgenic strain RW1596 *stEx30* [*myo-3p::gfp::myo-3+rol-6(su1006)*]. Animals were maintained on nutrient agar plates with *Escherichia coli* K12 prior to experiments. Nematode growth medium (NGM) plates containing Agar NO.1 (2.5% w/v, Oxoid), Pepton N-Z-Soy(R) BL4 (0.25% w/v, Sigma-Aldrich), NaCl (0.3% w/v), cholesterol (0.0005% v/v), CaCl_2_ (1 mM), MgSO_4_ (1 mM), K_2_HPO_4_/KH_2_PO_4_ (25 mM, pH 6.0), carbenicillin disodium (0.5 mg/ml, Fisher BioReagents) and isopropyl-β-D-thiogalactopyranoside (IPTG; Dioxane-free, 1 mM, Fisher BioReagents) were used for experiments. As future muscle integrity studies will rely mostly on the use of RNAi, we used the bacterial control strain *E. coli* HT115 containing the (empty) L4440 vector as food source. Bacteria were grown overnight on a shaker (120 rpm) at 37°C in LB Broth Lennox (2% w/v, Fisher BioReagents) containing carbenicillin disodium (2 mg/ml, Fisher BioReagents). IPTG (1 mM, Fisher BioReagents) was added the next day and the culture was placed on a shaker (120 rpm) for an additional 2 h at 37°C. Bacteria were washed with salt water (0.3% NaCl w/v) and concentrated five times. Seeded NGM plates were kept at 20°C to allow bacterial growth overnight. Synchronized L1 nematodes were spotted onto these plates and FUDR (100 µM, Acros Organics) was added at L4 stage to prevent offspring. Animals were transferred regularly to fresh plates during the experiment.

### Image acquisition

A Nikon TiE-C2 confocal laser-scanning microscope, equipped with a 40× WI Plan Apo objective (1.20 NA, water immersion) was used for imaging. Images (1024×512 pixels, 6.67 pixels/µM, scanner zoom 2×) were collected by exciting with a 488 nm solid-state argon laser and detecting through a 525/50 nm bandpass filter. Laser power (1.25%), gain (82) and offset (5) were maintained constant throughout all experiments. Images were collected for four independent ageing cohorts at specific time points during ageing (at day 1, 4, 6, 8, 12, 14 and 18 of adulthood). In all individuals, body wall muscle cells from the midsection of the body were imaged as these cells allow for imaging with minimal distortion. Standardized image nomenclature was used to facilitate downstream analysis. We recommend using the following format: C01W01M01.nd2, in which C, W and M refers to the condition, worm and myofilament, respectively. The total numbers of worms and muscle cells per condition are summarized in Table 1.

### Manual scoring of muscle deterioration

Images of body wall muscle cells were evaluated in a randomized and blinded manner by an expert scientist using Blinder freeware (Cothren et al., 2018). Each cell was assigned one of three qualitative classes of muscle deterioration: (1) normal, healthy cell with well-organized myofilaments, (2) moderately disorganized cell and (3) severely distorted cell.

### Image analysis

All image analysis was performed in FIJI open-source image processing software (Schindelin et al., 2012). A macro script (MuscleMetrics.ijm) was written for the assessment of muscle organization in *myo-3*::GFP worms, which is available at GitHub (https://github.com/DeVosLab/MuscleMetrics). The script consists of a set of interactive tools for cropping and aligning individual muscle cells as well as an automated routine for segmentation and morphological feature extraction of individual myofilaments (Fig. 1). In brief, the algorithm consists of local contrast enhancement, background subtraction and a directional second-order edge enhancement (using FeatureJ plugin; https://imagescience.org/meijering/software/featurej/), followed by binarization according to Yen's autothresholding algorithm (Yen et al., 1995). Subsequent particle analysis is done with exclusion of single pixel objects so as to remove noise contributions. Apart from the morphological and intensity metrics that are extracted by default in ImageJ, the analysis also includes measurement of local thickness and curvature variations. Local thickness descriptors (average, range, variation) are directly based on the output of the Local Thickness plugin (https://github.com/fiji/LocalThickness). Curvature is expressed as a deviation of the object's perimeter with respect to that of a reconstructed version based on a subset of (first five) elliptic Fourier descriptors using the Fourier Shape Analysis plugin (https://imagejdocu.tudor.lu/plugin/analysis/fourier_shape_analysis/start). Simple, smooth shapes will have a curvature value close to one, whereas irregular objects will have larger curvature values. Skeletonization of the binary objects provides additional info on the number of branch points and straightness (Feret/Area of the skeleton), whereas the smallest eigenvalues of the structure tensor (as obtained from FeatureJ) are used for calculating the bending energy and determining the number of bend points (local maxima). Finally, a global analysis of myofilament orientation is performed using the Directionality plugin (https://github.com/fiji/Directionality). Per muscle cell, images are stored along with the corresponding regions of interest (ROI) sets and result files.

### Statistics

All data analysis was performed in R Studio, using R version 4.0.2. Plotting was done using the ggplot2 package and summary statistics were calculated using the ddply package. All individual result files were loaded into a single data frame, collated with the appropriate metadata regarding experimental conditions (including factors such as age, worm, muscle and replicate). During image analysis, features were extracted at the individual myofilament level. To evaluate changes at the cellular level, the average as well as the covariance of these values were calculated per muscle cell. These secondary data were combined with features that are inherently derived on a per cell basis such as directionality parameters, global cell size and the total number of myofilaments. Linear models were established using the native lm functions, and collinearity checks were performed using the variance inflation (VIF) inspection tool of the car package as well as the correlation detection of the caret package. General linear models with smoothing terms were established using the mgcv package. Model performance was assessed in terms of the adjusted squared correlation (*R*^2^) and the RMSE between the predicted and observed values. To rescale the predicted value (*Y*) from the linear models to the actual age range of the worms (day 1-18), the following formula was used:

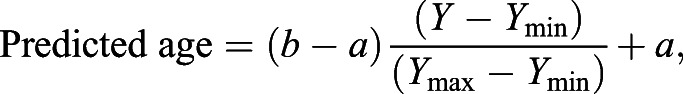
in which *Y*_min_ and *Y*_max_ represent the minimum and maximum average predicted value of the model, and *a* and *b* represent value 1 and value 18, respectively.

Statistical comparison of means was done using a Welch *t*-test after having verified for normality (Shapiro test and qq-plot) and homoscedasticity (Levene's test).

## Supplementary Material

Supplementary information
