# Supplementary material for: Prediction of biological age by morphological staging of sarcopenia in Caenorhabditis elegans
Source: Dis Model Mech. 2021 Nov 30;14(11):dmm049169. doi: 10.1242/dmm.049169 (PMC8649172; doi:10.1242/dmm.049169)
Supplement: Supplementary information [file dmm-14-049169-s1.pdf]

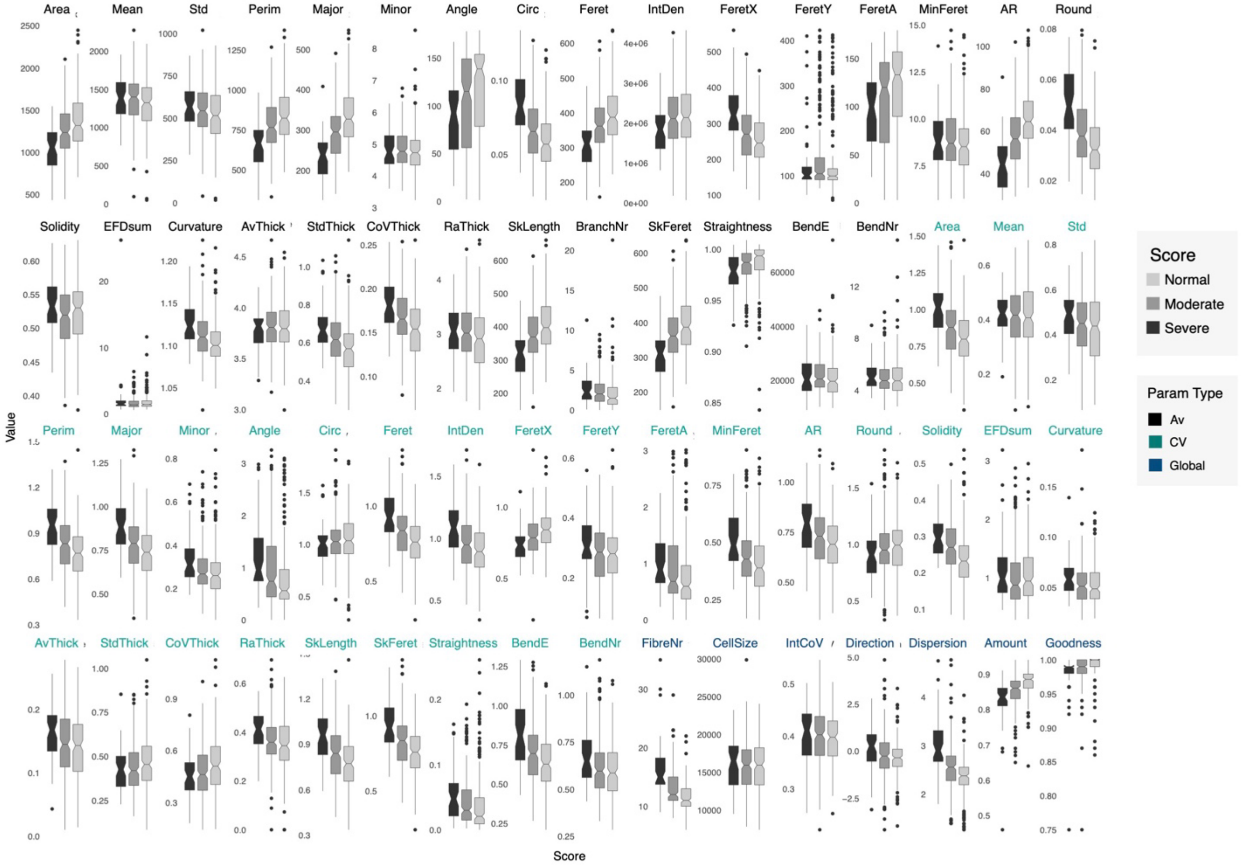

Fig. S1. Boxplots of all features as a function of manually assigned sarcopenia score.

Disease Models & Mechanisms • Supplementary information

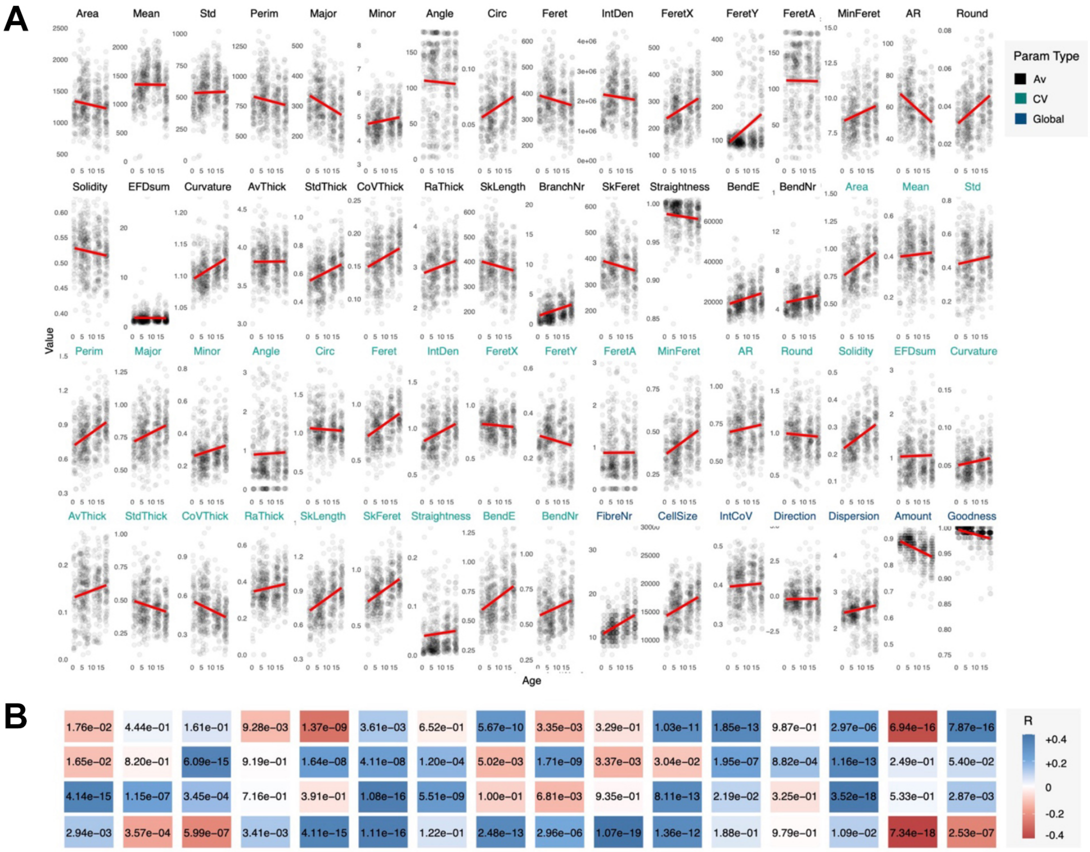

Fig. S2. Univariate regression of individual features as a function of chronological age. (A) Scatterplots of with linear regression line in red. (B) Color-coded correlation coefficients of the same parameters with their respective p-value.

Disease Models & Mechanisms • Supplementary information

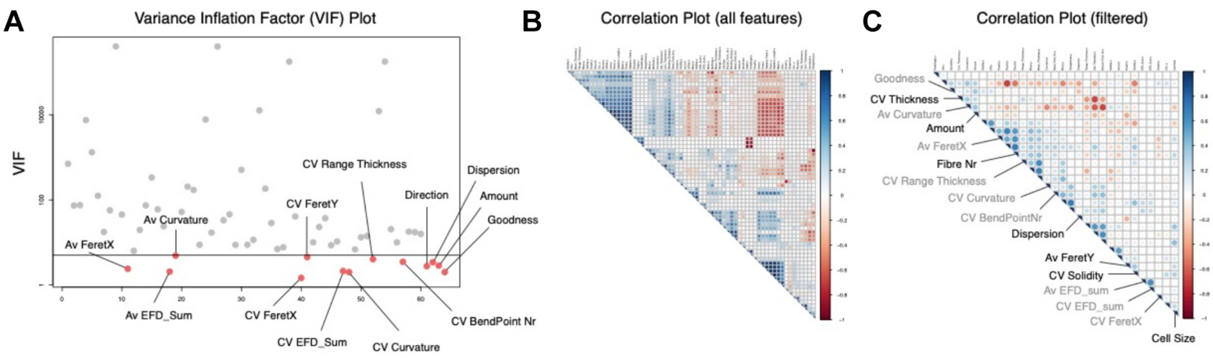

Fig. S3. Complementary feature selection methods for model simplification. (A) variance inflation factor (VIF) plot of the complete model showing features with a VIF below 5 in red. (B, C) Correlation plot of all features (B) and after retention of only those features with correlation below 0.7 (C). Those corresponding with VIF selected are indicated in grey, those retained in the final model in black.
